# Comparison between the Effects of Oral and Intramuscular Administration of Shin'iseihaito (Xinyiqingfeitang) in a* Streptococcus pyogenes*-Induced Murine Sinusitis Model

**DOI:** 10.1155/2018/8901215

**Published:** 2018-05-02

**Authors:** Masaaki Minami, Toru Konishi, Hiroshi Takase, Toshiaki Makino

**Affiliations:** ^1^Department of Bacteriology, Graduate School of Medical Sciences, Nagoya City University, 1 Kawasumi, Mizuho-ku, Nagoya, Japan; ^2^Department of Pharmacognosy, Graduate School of Pharmaceutical Sciences, Nagoya City University, 3-1 Tanabe-dori, Mizuho-ku, Nagoya, Japan; ^3^Core Laboratory, Graduate School of Medical Sciences, Nagoya City University, 1 Kawasumi, Mizuho-ku, Nagoya, Japan

## Abstract

*Streptococcus pyogenes (S. pyogenes)* is a species of Gram-positive coccoid bacteria having many virulence factors. Its capsule and exotoxins can cause upper respiratory tract infections such as sinusitis. The general treatment for* S. pyogenes*-induced sinusitis is administration of antibiotics such as penicillin and macrolides; however, a serious problem associated with these antibiotics is their attenuated effect. Shin'iseihaito (Xinyiqingfeitang), a formula of Japanese traditional Kampo medicine and traditional Chinese medicine, has been used for the treatment of sinusitis. In general, formulas of Japanese traditional Kampo medicine are orally administered. This is in contrast to certain formulas of traditional Chinese medicine, which are being recently administered intramuscularly or intravenously. Regarding these traditional Chinese medicine formulas, the injection methodology is reported to be more effective than oral intake. In this study, we compared the efficacy between orally and intramuscularly administered Shin'iseihaito against* S. pyogenes*-induced sinusitis. We evaluated the antibacterial effect of Shin'iseihaito extract (SSHT) against* S. pyogenes *by K-B disk diffusion assay. Furthermore, we investigated the nasal colonization of* S. pyogenes*, determined cytokine (TNF-*α*, IL-1*β*, and IL-6) levels, and conducted a splenocyte proliferative assay in a murine sinusitis model. SSHT displayed direct anti-*S. pyogenes* activity. Intramuscular administration of SSHT decreased the nasal colonization of* S. pyogenes* compared with oral administration. Thymidine uptake analysis revealed that the proliferation of splenocytes from* S. pyogenes*-infected mice under intramuscular SSHT treatment was upregulated compared to that of splenocytes from* S. pyogenes*-infected mice under oral SSHT treatment. We also found that TNF-*α*, IL-1*β*, and IL-6 levels in the nasal discharge from intramuscularly treated* S. pyogenes*-infected mice were lower than those from orally treated mice. Our findings suggest that intramuscular administration of Shin'iseihaito may be useful for the treatment of murine* S. pyogenes*-induced sinusitis.

## 1. Introduction

*Streptococcus pyogenes (S. pyogenes)* are Gram-positive bacteria that cause numerous human diseases including sinusitis, pharyngitis, tonsillitis, otitis media, cellulitis, necrotizing fasciitis, and streptococcal toxic shock syndrome [1]. Although antibiotics such as penicillin have been effective in treating* S. pyogenes* for a long time, there have been reports of several serious issues associated with this therapy [2], one of them being the attenuation of the antibiotic effect [3]. Additionally, there has also been a recent rise in macrolide-resistant* S. pyogenes* in Japan and across the world [4]. As* S. pyogenes* evolves gradually to gain resistance against other antibiotics, there is a pertinent need for new anti-infective therapies.

Sinusitis is a common disease characterized by persistent inflammation of the sinuses, which consists of the nasal and the paranasal sinus mucosa [5]. Most acute cases arise from bacterial and viral infections [6]. Previous studies have shown that, in response to these infections, cytokines play roles in initiating and maintaining inflammation, a condition characterized by neutrophil tissue infiltration [6].

Traditional Chinese medicine (TCM) is one of the most familiar alternative complementary therapies worldwide [7]. It is increasingly becoming well known in the field of otorhinolaryngology, where it is used to treat sinusitis and tinnitus [8]. In Japan, Kampo medicine, which has been developed from ancient Chinese medicine, is also recognized as an effective alternative medicine against several diseases [9]. Shin'iseihaito (Xinyiqingfeitang) is a formula containing 9 crude drugs from both traditional Japanese Kampo medicine and TCM. In Japan, it has been used for the treatment of upper respiratory tract diseases, especially sinusitis [10]. Clinical investigation of the characteristics of adjunctive TCM use in patients with chronic rhinosinusitis showed that the most common Chinese herbal formula used was Xinyiqingfeitang in Taiwan [11]. In our previous studies, we investigated the antibacterial activity of the Shin'iseihaito extract (SSHT) against* S. pneumoniae in vitro* and the preventive effect of SSHT in an ovalbumin-induced allergic rhinitis model [12–15]. In principle, most Kampo formulas are orally administered in Japan. However, in China, it has been reported that these formulas are more effective when administered subcutaneously, intramuscularly, and intravenously. Further, some researchers have reported that injecting danhong and xuebijing was effective and useful in an animal experimental model [16, 17]. As nonoral administration of drugs/therapies by western medicine has been proven to have a better therapeutic effect, the nonoral administration of TCM is also expected to improve its therapeutic potential. We used a murine mouse model infected with* S. pyogenes* and employed a novel intramuscular administration method of SSHT to evaluate its effects against infection. In the present study, we report the efficacy of intramuscular SSHT against* S. pyogenes*-induced infection in a mouse sinusitis model.

## 2. Materials and Methods

### 2.1. Bacterial Strains

*Streptococcus pyogenes (S. pyogenes)* (DD328) was isolated from nasal discharge of a sinusitis patient in Japan. A fresh colony was inoculated overnight on TSAII sheep blood agar and cultured for 16 h at 37°C under 5% CO_2_. The bacteria were harvested, centrifuged, and resuspended in sterile phosphate-buffered saline (0.15 M, pH 7.2, PBS). Bacterial density was determined by measuring absorbance at 600 nm. The bacterial suspension was then diluted with PBS to 10^9^ CFU (colony forming unit)/mL using a standard growth curve to relate measured A600 to bacterial concentration.

### 2.2. Crude Drugs

Shin'iseihaito (Xinyiqingfeitang) (daily dose for human) consists of 3.0 g of the rhizome of* Anemarrhena asphodeloides*, 1.0 g of the rhizome of* Cimicifuga heracleifolia*, 2.0 g of the leaf of* Eriobotrya japonica*, 5.0 g of* Gypsum fibrosum*, 3.0 g of the fruit of* Gardenia jasminoides*, 3.0 g of the bulb of* Lilium lancifolium*, 3.0 g of the flower of* Magnolia salicifolia*, 5.0 g of the tuber of* Ophiopogon japonicas*, and 3.0 g of the root of* Scutellaria baicalensis*. These crude drugs were purchased from Daikoshoyaku (Nagoya, Japan) or Tsumura (Tokyo, Japan) and were standardized by Japanese Pharmacopoeia 17th Edition [18]. The mixture of above crude drugs was boiled in 20 times the weight of water for 30 min and filtered. The decoction was lyophilized to yield powdered extract (SSHT, 7.1 g for daily human dose). Fingerprint pattern of this SSHT was shown in our previous study [13]. SSHT was suspended in distilled water to prepare the stock solution at a concentration of 0.1 g/mL and kept in −20°C until use.

### 2.3. Disk Diffusion Assay

Antibacterial activity was evaluated by the disk diffusion method as described previously [13]. Sterile paper disks were infused with the SSHT extract (7.5 mg/disk) and dried at 25°C. Kirby-Bauer disk “EIKEN” containing penicillin G (10 U = 6 *μ*g/disk) was used as the positive control. The paper disk with PBS was used as the negative control. The colonies of* S. pyogenes* were cultured overnight on TSAII sheep blood agar at 37°C under 5% CO_2_ atmosphere and collected in Columbia broth (3.5 g/L) till a final density of 2.0 × 10^9^ was obtained. This bacterial suspension was then spread onto fresh TSAII sheep blood agar plates. This was followed by placing the paper disks onto these plates and incubating the plates at 37°C under 5% CO_2_ atmosphere for 20–24 h. Finally, the diameter of the inhibitory zone was measured.

### 2.4. Bacterial Morphologic Investigation

Bacterial morphological analysis using transmission electron microscopy JEM1011J was carried out as described earlier [19]. The* S. pyogenes* strains treated with SSHT (0, 0.5, and 5 mg/mL) were cultured for a day in Todd Hewitt medium with 5% yeast extract. For negative staining, approximately one drop of the bacterial culture was applied onto a 300-mesh carbon formvar copper grid. Excess solution was removed, and negative staining was performed using 2% phosphotungstic acid. The samples were then observed by electron microscopy, and digital images were captured using a MegaView slow-scan camera (JEOL).

### 2.5. Murine Model of Nasal Infection

**Figure 1:**
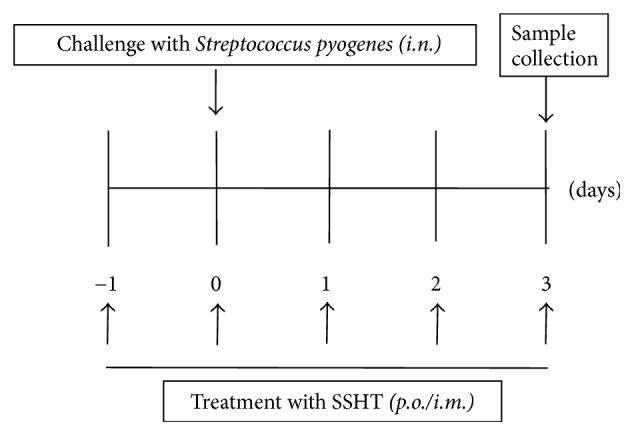
Schematic describing the protocols used for experiments with the* S. pyogenes*-induced murine sinusitis model. In the infected group, 1 × 10^7^ CFU bacteria were injected into both nostrils of mice using a 29-gauge needle at day 0. Shin'iseihaito extract (SSHT) or phosphate-buffered saline (PBS) control was orally* (p.o.)* or intramuscularly* (i.m.)* administered daily from day 0 to day 3.

The effect of SSHT on the* S. pyogenes*-induced murine sinusitis model after nasal inoculation was assessed using the following procedure [15].* S. pyogenes* were cultured for 16 h on TSAII sheep blood agar, harvested, and mixed in 1 mL of PBS followed by centrifugation at 2,000 ×g for 2 min. The pellets were diluted in 100 *μ*L PBS to a final density of 1 × 10^9^ CFU and then inoculated into both nostrils of inbred 4-week-old male ICR mice using a 29-gauge needle. The number of CFUs inoculated was verified for each experiment by plating the bacteria on TSAII sheep blood agar and counting CFUs. Mice were observed daily. In the SSHT-treated group, mice were given SSHT on days −1, 0, 1, 2, and 3 after* S. pyogenes* inoculation either orally or intramuscularly (Figure 1). The SSHT doses were 2.9 g/kg body weight (b.w.), which is 20 times the human daily dose, or 0.29 g/kg (b.w.). The mice in the control group were given PBS. As a positive control, ampicillin sodium (100 mg/kg) was administered intramuscularly. Each experiment consisted of 3 mice per group, and with 2 independent experiments, a total of 6 mice were studied.

### 2.6. Nasal Lavage Analysis

Procedures to obtain nasal cultures have been described previously [15]. In brief, the mice were sacrificed by CO_2_ inhalation. Subsequently, the external nares, oral cavity, and head were disinfected with a moist alcohol swab and allowed to dry. Nasal lavage was performed with 200 *μ*L of PBS. The recovered fluid was then serially diluted, and 10 *μ*L of each dilution was plated onto TSAII sheep blood agar plates. The plates were incubated for 24 h, and then colonies of* S. pyogenes *were counted. The results were quantified as the number of CFUs/mL. Furthermore, by using the aliquot of nasal lavage, the concentrations of tumor necrosis factor-alpha (TNF-*α*), interleukin-1*β* (IL-1*β*), and IL-6 were measured using enzyme-linked immunosorbent assay (ELISA) kits (BioLegend, San Diego, CA, USA).

### 2.7. Determination of Splenocyte Proliferative Response

After the mice were sacrificed by CO_2_ inhalation, the spleen was removed aseptically, and splenocytes were filtered and cultured in RPMI 1640 with 5% fetal calf serum medium. At 20 h prior to the culmination of the splenocyte culture, ^3^H-thymidine (2.0 Ci/mmol; PerkinElmer, MA, USA) was added to the medium. Once culturing was completed, the cells were adsorbed on 0.45 *μ*m membrane filters, washed with distilled water, and then dried. The filters were transferred to vials filled with liquid scintillator cocktail, and the radioactivity was measured by using a liquid scintillation counter (LSC-6100, Hitachi Aloka Medical, Tokyo, Japan). Results are given as DPM (Disintegrations per Minute).

### 2.8. Statistical Analysis

All statistical analyses were conducted using Tukey's multiple comparison test for differences among multiple groups (EZR version 1.36). Values less than 0.05 indicated statistical significance.

## 3. Results

### 3.1. Disk Diffusion Assay

**Figure 2:**
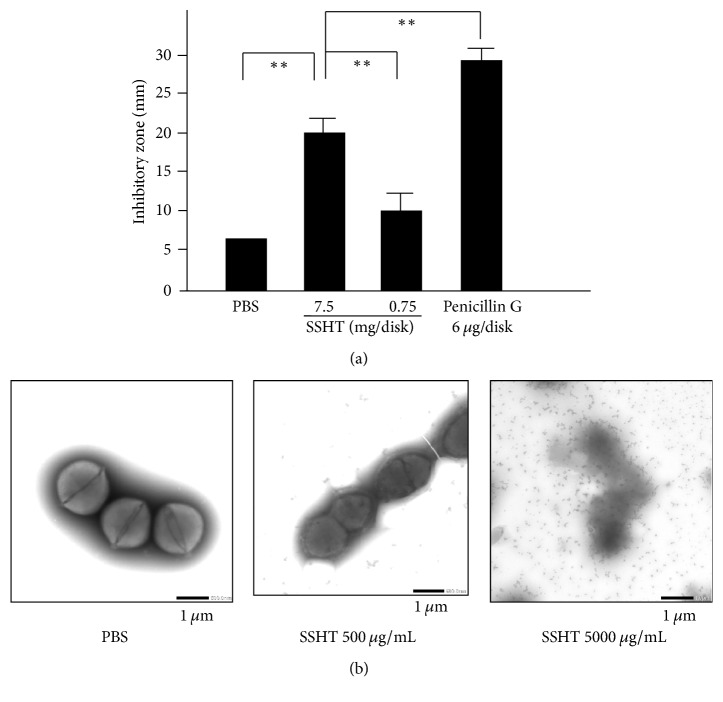
(a) Antibacterial effect of Shin'iseihaito extract (SSHT) against* S. pyogenes.* The diameter of the inhibitory zone after 18–24 h of incubation was measured. Values are expressed as the mean ± SD (*n* = 6). ^*∗∗*^*p* < 0.01. (b) Morphological changes in* S. pyogenes* treated with SSHT or phosphate-buffered saline (PBS). Representative images of* S. pyogenes* treated with SSHT for 1 day using electron microscopy and negative staining.

Antibacterial activity of the SSHT extract against* S. pyogenes* was evaluated by using the disk diffusion method. As shown in Figure 2(a), the SSHT extract exhibited antibacterial activity against* S. pyogenes* in a dose-dependent manner. However, the SSHT extract (7.5 mg/disk) displayed a lower antibacterial activity than penicillin G (6 *μ*g/disk).

### 3.2. Bacterial Morphology

We tried to assess the direct effect of SSHT against* S. pyogenes* by morphological analysis. Negative staining analysis revealed that SSHT-treated bacteria were susceptible, displaying a decline in morphological integrity in a dose-dependent manner (Figure 2(b)).

### 3.3. Murine Nasal Colonization

**Figure 3:**
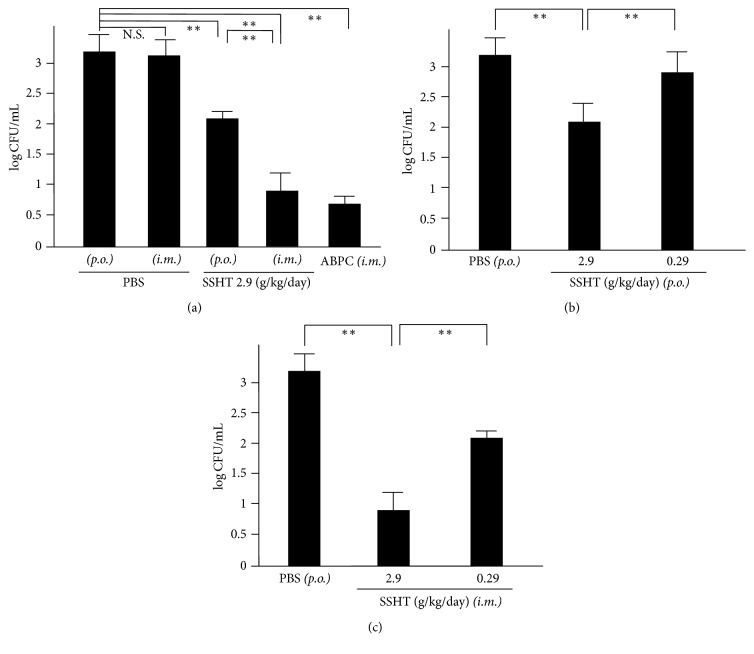
The colonies of* S. pyogenes* in nasal lavage collected from Shin'iseihaito extract- (SSHT-) treated mice). Mice were treated with SSHT according to the protocol shown in Figure 1. The nasal fluids were inoculated on TSAII sheep blood agar and incubated for 24 h. (a) Comparison of colony counts among phosphate-buffered saline (PBS) oral* (p.o.)*, PBS intramuscular* (i.m.)*, SSHT oral* (p.o.)*, SSHT intramuscular* (i.m.)*, or ampicillin sodium (100 mg/kg/day* (i.m.)*, ABPC) treatment groups. (b) Dose-dependent effect of* p.o.* SSHT or (c)* i.m.* SSHT on nasal* S. pyogenes* infection. Data represent the mean ± SD (*n* = 6). N.S.: not significant. ^*∗∗*^*p* < 0.01.

We tried to assess whether SSHT provided protection against* S. pyogenes* nasal infection and, if so, which of the oral and the intramuscular administration of SSHT was more effective. Our studies showed that, three days after infection with* S. pyogenes*, the CFUs count in the nose of oral SSHT-treated mice was significantly lower than that in the nose of SSHT-untreated mice (*p* < 0.01) (Figure 3(a)). We confirmed that both oral and intramuscular SSHT administrations reduced* S. pyogenes* colony formation in the murine nose in a dose-dependent manner (Figures 3(b) and 3(c)). This finding highlights the protective role of SSHT against* S. pyogenes* nasal infection. We further observed a significant drop in the CFUs count in the nose of intramuscular SSHT-treated mice compared to that in the nose of oral SSHT-treated mice (*p* < 0.01). This suggests that SSHT administered intramuscularly was more effective in fending off* S. pyogenes* nasal infection than SSHT that was orally administered. We noted no significant difference between the oral and intramuscular administrations of PBS; therefore, subsequent experiments were carried out using only orally administered PBS as a control.

### 3.4. Cytokine Expression in Nasal Lavage from SSHT-Treated Mouse

**Figure 4:**
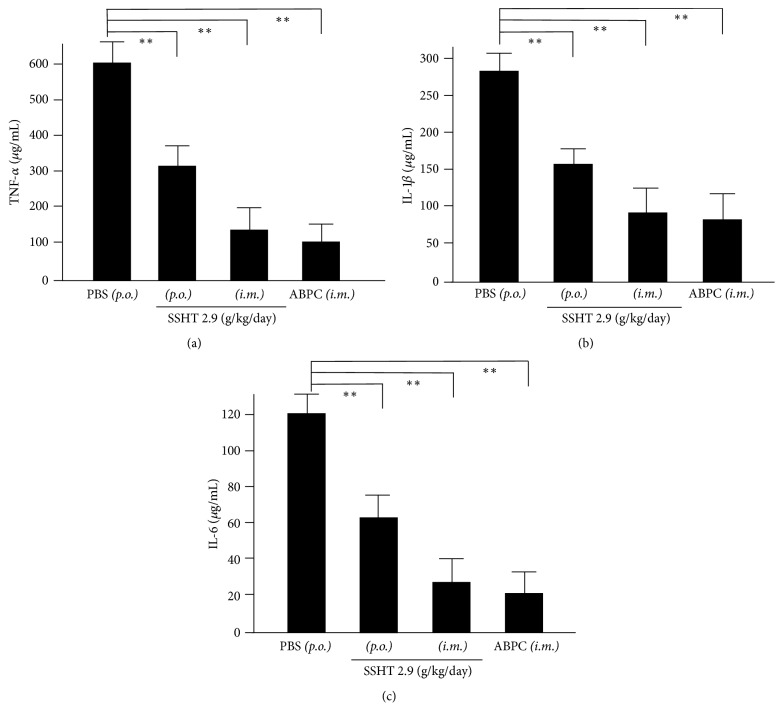
Cytokine levels in nasal lavage collected from Shin'iseihaito extract- (SSHT-) treated mice. Mice were treated with SSHT, phosphate-buffered saline (PBS)* (p.o.)*, or ampicillin sodium (100 mg/kg/day, ABPC)* (i.m.)* according to the protocol shown in Figure 1. The nasal fluids were collected and TNF-*α* (a), IL-1*β* (b), and IL-6 (c) levels were measured by using ELISA. Data represent the mean ± SD (*n* = 6). ^*∗∗*^*p* < 0.01.

We next examined cytokine expression in nasal lavage collected following SSHT treatment. The levels of TNF-*α*, IL-1*β*, and IL-6 in murine nasal lavage were measured by ELISA. Our results showed that the levels of these cytokines were significantly downregulated in mice treated with oral SSHT compared to those in untreated mice (*p* < 0.01) (Figure 4). The levels of these cytokines were significantly suppressed in mice treated with either oral or intramuscular SSHT compared to those in control mice (*p* < 0.01). We also noted that the levels of these cytokines were further suppressed in animals treated with intramuscular SSHT than in those treated orally.

### 3.5. Splenocyte Proliferative Activity in SSHT-Treated Mice

**Figure 5:**
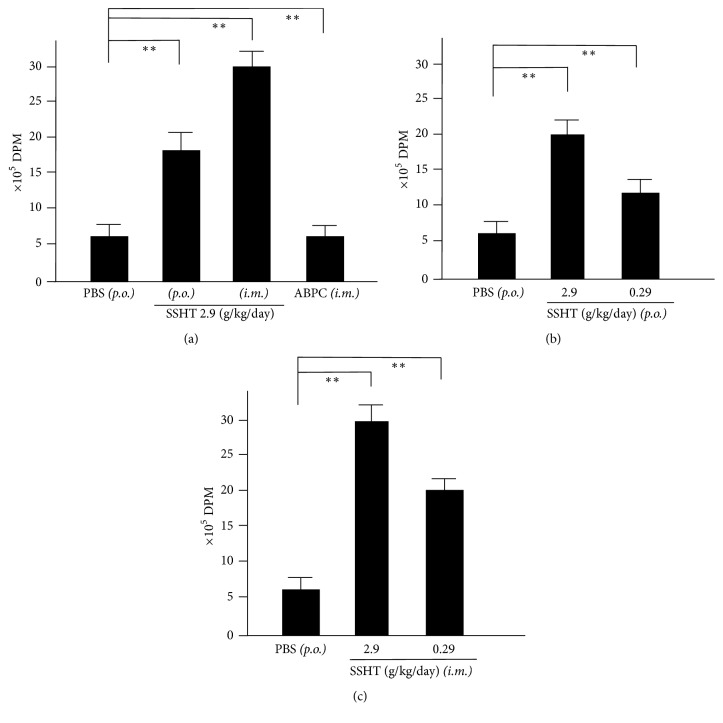
^3^H-thymidine-uptake assay of splenocytes collected from Shin'iseihaito extract- (SSHT-) treated Mice. Mice were treated with SSHT, phosphate-buffered saline (PBS)* (p.o.)*, or ampicillin sodium (100 mg/kg/day, ABPC)* (i.m.)* according to the protocol shown in Figure 1, and then splenocytes were collected. (a) Comparison of splenocyte proliferation among PBS oral* (p.o.)*, PBS intramuscular* (i.m.)*, SSHT oral* (p.o.)*, SSHT intramuscular* (i.m.)*, or ampicillin sodium (100 mg/kg/day* (i.m.)*, ABPC) treatment groups. (b) Dose-dependent effect of* p.o.* SSHT (c) and* i.m.* SSHT treatments on nasal* S. pyogenes* infection. Data represent the mean ± SD (*n* = 6). ^*∗∗*^*p* < 0.01.

Finally, we studied splenocyte activity, because splenocytes play a major role in murine bacterial infection models. To determine whether splenocytes from SSHT-treated mice showed elevated activity, we performed ^3^H-thymidine uptake analysis. As shown in Figure 5(a), the uptake of ^3^H-thymidine into splenocytes collected from mice orally treated with SSHT was significantly higher than that from untreated mice. Furthermore, ^3^H-thymidine uptake in mice intramuscularly injected with SSHT was significantly higher than that from mice orally given SSHT. Finally, we confirmed that both oral and intramuscular SSHT administrations upregulated the uptake of ^3^H-thymidine in a dose-dependent manner (Figures 5(b) and 5(c)).

## 4. Discussion

To our knowledge, this is the first experimental study in which intramuscular SSHT injection has been shown to be effective in a* S. pyogenes*-induced murine sinusitis model. Our results showed that SSHT regulates cytokine responses more effectively when administered intramuscularly than orally to protect against* S. pyogenes.* Our investigation contributes toward advancement in using western science-based methodologies to assess the potential activity of Japanese/Chinese natural products.

We first evaluated the direct anti-*S. pyogenes* effect of SSHT by using the K-B disk analysis and negative staining assay. Mice intramuscularly treated with SSHT after nasal* S. pyogenes* infection showed significant reduction in* S. pyogenes* colonization in the nose along with upregulation in splenocyte proliferative activity and suppression of excess cytokine production, including TNF-*α*, IL-1*β*, and IL-6, compared with mice orally treated with SSHT. These results suggest that intramuscular SSHT injection may be instrumental in protection against* S. pyogenes*-induced murine sinusitis. The injections are new dosage forms of TCM in China, developed from the traditional preparations [20]. Unlike oral administration, most Chinese medicines are administered intramuscularly for the treatment of various diseases. Injecting drugs facilitates their rapid distribution in target tissues and receptors bringing about a quick onset [20]. Several reports have highlighted the advantage of this aspect of injected drugs in time-sensitive severe cases including rescue and emergency [16, 17]. Our experimental results further add to these findings and may partially explain the benefit of TCM injections.

Regarding the consequence of direct drug administration to treat bacterial infections, a previous study has shown that baicalin, an ingredient of the* Scutellariae Radix* root extract, demonstrated remarkable inhibitory effect against* S. pneumoniae *bacterial growth [12]. From this result, there may be a probability that the antimicrobial mechanism of action of baicalin could also extend to* S. pyogenes, *streptococci similar to* S. pneumoniae*.

Nasal lavage collected from intramuscular SSHT-treated mice showed significantly lower TNF-*α*, IL-1*β*, and IL-6 levels than those from oral SSHT-treated mice. In this study, we focused on inflammatory cytokines (TNF-*α*, IL-1*β*, and IL-6). These inflammatory cytokines that activate immunological activity are induced by bacterial infections and cause inflammation [6]. Although the relationship between inflammatory cytokines and* S. pyogenes*-induced sinusitis has not been studied precisely, a previous report identified* S. pneumoniae-*induced inflammatory cytokines in bronchoalveolar lavage fluid samples from a mouse pneumonia model [21]. Our results are partially consistent with previous reports and suggest that SSHT-induced cytokine production augments bactericidal activity including immunomodulation that causes bacterial elimination.

Activation of splenocytes by SSHT may induce activation of immune function, which may be more effective for intramuscular injections as with vaccinations. However, intramuscular injection of more potent immunologically active herbal medicine may cause an adverse response such as an excessive immune reaction [22]. Thus, further studies on safety of the host in future experiments using animals and humans are necessary.

Although some herbal medicines activate immune function, they also function to suppress excessive immune reaction [22–25]. Our results also show that SSHT behaves in this manner by suppressing excessive immune responses while activating the necessary immune response. Our data also hint at SSHT being useful in the treatment of multidrug-resistant* S. pyogenes *[2]. Despite SSHT having an antibacterial effect, its effective optimum concentration is considerably high compared to those of commercially available western antibiotics. Therefore, SSHT is not per se an antibiotic but more of an immunomodulatory agent. Together, our results imply that SSHT affects the host's immune system to mount an immune response when infected by* S. pyogenes*.

Although Kampo medicines have immunomodulatory effects, such as activation of splenocytes, the details of their mechanisms of action are unknown. Kampo medicines are generally composed of several crude drug components, and the interaction between them may enhance their overall medicative and therapeutic effect. Although further investigation from these perspectives is needed, SSHT has shown potent immunomodulatory effects. Finally, further studies involving human subjects investigating the effects of intramuscular SSHT administration against* S. pyogenes* and other bacterial infections would be extremely beneficial to develop novel antibiotic strategies.

In summary, Shin'iseihaito is significantly effective in the treatment of* S. pyogenes-*induced sinusitis in a murine model. We also suggest intramuscular Shin'iseihaito administration as a therapeutic candidate for effective treatment against complicated human sinusitis induced by* S. pyogenes*.
